# Menhaden oil, but not safflower or soybean oil, aids in restoring the polyunsaturated fatty acid profile in the novel delta-6-desaturase null mouse

**DOI:** 10.1186/1476-511X-11-60

**Published:** 2012-05-29

**Authors:** Jessica Monteiro, Feng-Jun Li, Mira MacLennan, Alexandra Rabalski, Mohammed H Moghadasian, Manabu T Nakamura, David WL Ma

**Affiliations:** 1 Department of Human Health and Nutritional Sciences, University of Guelph, Guelph, ON, N1G 2W1, Canada; 2 Department of Human Nutritional Sciences, University of Manitoba, Winnipeg, MB, R2H 2A6, CAnada; 3 Department of Food Science and Human Nutrition, Division of Nutritional Sciences, University of Illinois at Urbana-Champaign, Urbana, IL, 61801, USA

**Keywords:** Knock-out, Liver, Alpha-linolenic acid, Linoleic acid, HUFA, Delta-6 desaturase knock-out, Delta-5 desaturase

## Abstract

**Background:**

Polyunsaturated fatty acids (PUFA) have diverse biological effects, from promoting inflammation to preventing cancer and heart disease. Growing evidence suggests that individual PUFA may have independent effects in health and disease. The individual roles of the two essential PUFA, linoleic acid (LA) and α-linolenic acid (ALA), have been difficult to discern from the actions of their highly unsaturated fatty acid (HUFA) downstream metabolites. This issue has recently been addressed through the development of the Δ-6 desaturase knock out (D6KO) mouse, which lacks the rate limiting Δ-6 desaturase enzyme and therefore cannot metabolize LA or ALA. However, a potential confounder in this model is the production of novel Δ-5 desaturase (D5D) derived fatty acids when D6KO mice are fed diets containing LA and ALA, but void of arachidonic acid.

**Objective:**

The aim of the present study was to characterize how the D6KO model differentially responds to diets containing the essential n-6 and n-3 PUFA, and whether the direct provision of downstream HUFA can rescue the phenotype and prevent the production of D5D fatty acids.

**Methodology:**

Liver and serum phospholipid (PL) fatty acid composition was examined in D6KO and wild type mice fed i) 10% safflower oil diet (SF, LA rich) ii) 10% soy diet (SO, LA+ALA) or iii) 3% menhaden oil +7% SF diet (MD, HUFA rich) for 28 days (n = 3-7/group).

**Results:**

Novel D5D fatty acids were found in liver PL of D6KO fed SF or SO-fed mice, but differed in the type of D5D fatty acid depending on diet. Conversely, MD-fed D6KO mice had a liver PL fatty acid profile similar to wild-type mice.

**Conclusions:**

Through careful consideration of the dietary fatty acid composition, and especially the HUFA content in order to prevent the synthesis of D5D fatty acids, the D6KO model has the potential to elucidate the independent biological and health effects of the parent n-6 and n-3 fatty acids, LA and ALA.

## Background

Δ6-desaturase (D6D) is the first enzyme in the metabolic conversion of α-linolenic acid (ALA), an n-3 polyunsaturated fatty acid (PUFA), to eicosapentaenoic acid (EPA), and docosahexaenoic acid (DHA). D6D is also responsible for initiating the conversion of the n-6 PUFA, linoleic acid (LA), to arachidonic acid (AA). D6D is encoded by the FADS2 gene which is present in all mammals [1] and is most highly expressed in the liver [2], the main site of PUFA metabolism [3-5]. While mammals can endogenously produce a limited amount of AA, EPA and DHA from dietary LA and ALA, mammals do not have the ability to produce LA/ALA endogenously. Thus, both these fatty acids must be attained solely through dietary means and are considered essential fatty acids (EFA) [6].

Highly unsaturated fatty acids (HUFA), such as AA, EPA and DHA are primarily deposited in membrane phospholipids (PL) as opposed to intracellular triglyceride stores, and contribute to a wide array of cellular functions such as eicosanoid synthesis, gene expression [7], cell signaling, and protein function [8]. Liver phospholipids are differentially enriched in select fatty acids; phosphatidylcholine (PC) is the main PL and therefore contains the greatest quantity of fatty acids, while hepatic phosphatidylinositol (PI) and phosphatidylethanolamine (PE) are enriched in the eicosanoid precursor AA [9], and lyso-phosphatidylcholine (lyso-PC) is preferentially enriched with DHA for delivery to the brain [10].Thus, it is of interest to determine the fatty acid composition of each PL fraction in order to fully explore the effects of perturbations in essential fatty acid metabolism.

Select n-6 and n-3 PUFA have garnered attention from the scientific community and general public for their roles in the prevention and treatment of lifestyle diseases such as cardiovascular disease, Type II diabetes and certain cancers. Many studies [11-13] have aimed to assess the protective or therapeutic effects of either ALA or LA through dietary intervention. However, these studies cannot attribute results directly to either ALA or LA as there is always some conversion to more highly unsaturated fatty acids (HUFA), such as AA, EPA and DHA. Thus, any outcomes observed in such studies could also be due to the effects of AA, EPA and DHA which are all considered biologically active metabolites. Similarly, studies aiming to determine the effects of restricting a particular HUFA such as DHA or AA have not been able to do so without simultaneous removal of ALA or LA from the diet [14,15]. A model in which the metabolism of ALA/LA is inhibited would then be of great utility in determining if LA and ALA are independently able to prevent or alleviate disease.

**Figure 1:**
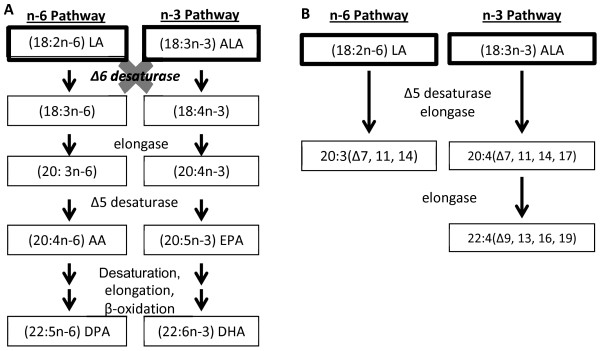
**A) The normal n-6 and n-3 fatty acid metabolic pathway. B)** In the absence of Δ6 desaturase, Δ5 desaturase and elongases metabolize LA and ALA to produce 3 novel fatty acids: 20:3(Δ7, 11, 14); 20:4(Δ7, 11, 14, 17), and 22:4(Δ9, 13, 16, 19).

This hurdle has recently been addressed with the development of the novel Δ-6 desaturase knock out (D6KO) mouse [16,17]. The homozygous D6KO mouse lacks a functional copy of the FADS2 gene, and is therefore unable to produce the D6D protein (Figure 1). This renders the D6KO mouse completely deficient in EPA/DHA/AA if these HUFA are not provided in the diet, since no alternative pathway for the production of these long chain fatty acids exists.

Previous studies with the D6KO mouse have focused on the effects of HUFA restriction through the provision of LA and ALA-enriched soybean oil on macrophage activity [18], dermatitis, and intestinal ulceration [17]. In contrast, the effects of a diet enriched only in LA on PUFA metabolism and HUFA synthesis in the D6KO mouse are currently unknown. Dietary studies have also been carried out on D6KO mice using diets enriched with purified AA (ARASCO) and DHA (DHASCO) oils [15,17]. However, the effect of HUFA-enriched oils more commonly found in human diets, such as fish oil, have not been explored in this novel mouse model.

The objective of the current study was to determine the differential effects of LA and ALA-enriched diets in D6KO mice**,** with special focus on phospholipid PUFA composition in the liver, the primary site of PUFA metabolism. Secondly, to evaluate if adding a natural source of HUFA (menhaden oil) to the diet can effectively restore the hepatic phospholipid HUFA content of D6KO mice **t**o levels seen in wild-type (WT) mice. Results of this study further support the use of the D6KO model for future studies aiming to determine the independent effects of LA and ALA in ameliorating disease.

## Results

### Animals

Mean mouse body weight and food intake across all treatment groups was not significantly different at the end of the study (Table 1). Data derived from D6KO groups were compared directly with WT groups, as heterozygous D6KO hepatic PL profiles did not differ from WT liver PL profiles.

**Table 1:** Mean weight gain and diet consumption

**Diet**	**Genotype**	**n**	Mean Weight Gain (g)*	Mean Diet Consumed (g)*
**Safflower**	WT	7	6.2 ± 1.9	71.8 ± 4.6
	KO	7	4.7 ± 1.7	70.16 ± 5.45
**Soy**	WT	5	4.6 ± 2.2	71.3 ± 5.7
	KO	3	5.1 ± 1.0	71.6 ± 6.0
**Menhaden**	WT	4	6.6 ± 1.6	75.6 ± 4.1
	KO	1	5.6 ± 1.9	78.9**

Note: *Weight gain is calculated from day on which mice were placed on experimental diet until day mice were terminated. Diet consumption was calculated from day mice were placed on experimental diet to Day 25.**Diet consumption data available for one mouse.

### The production of D5D novel fatty acids and their selective distribution in the liver

**Figure 2:**
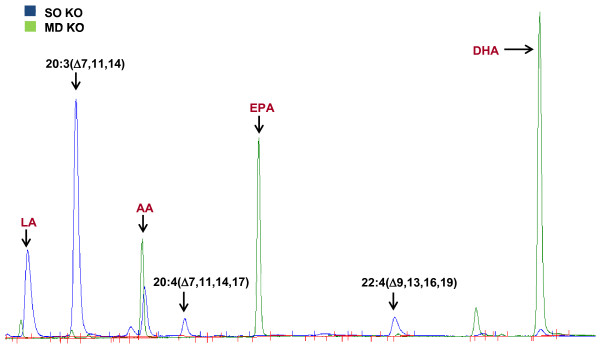
**Novel Δ-5 desaturase (D5D) products are found in soybean oil (SO) fed D6KO mice.** Representative chromatogram of the PC fraction obtained from liver samples of SO fed D6KO sample (BLUE) superimposed on a MD-fed D6KO (GREEN). D5D products [20:3(Δ7,11,14); 20:4(Δ7,11,14,17); 22:4(Δ9,13,16,19)] are noted in black, major HUFA are noted in red.

**Figure 3:**
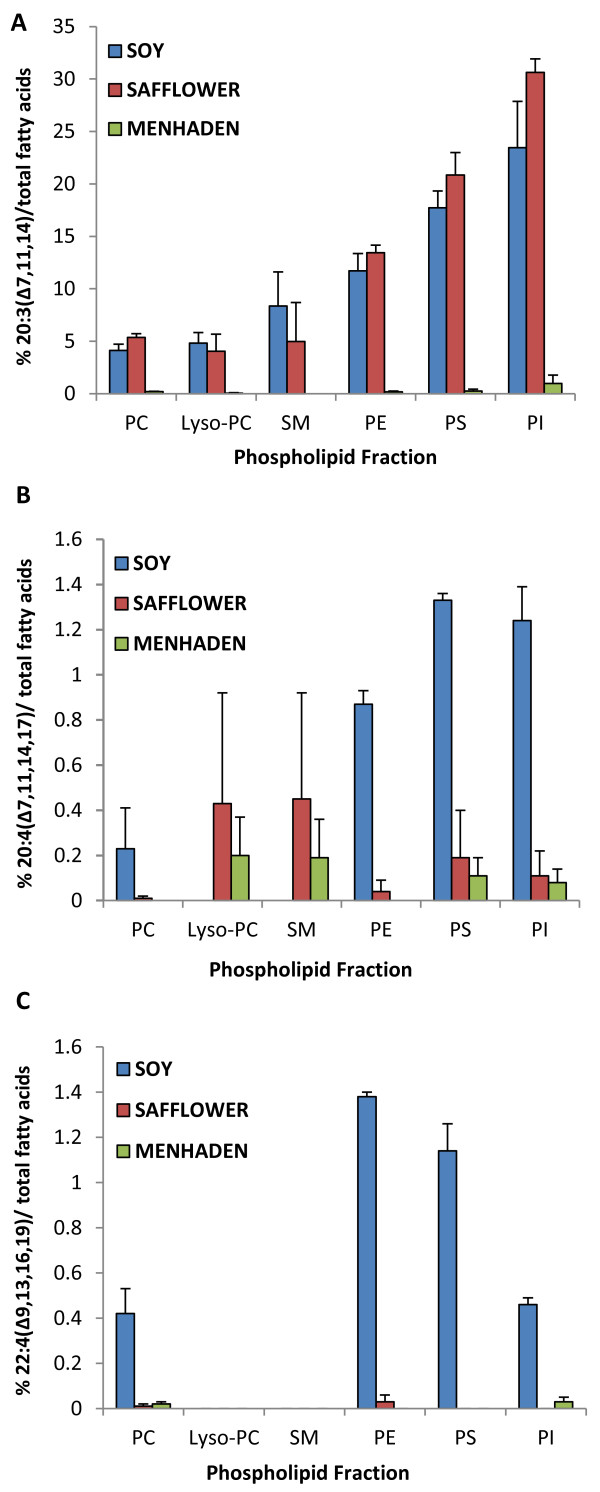
**Preferential distribution of 3 novel D5D fatty acids among the liver phospholipid fractions of D6KO mice across dietary treatment groups. A**) Distribution of 20:3 (Δ7,11,14); **B**) distribution of 20:4 (Δ7,11,14,17); **C**) distribution of 22:4 (Δ9,13,16,19).

Based on previously published results [17], three peaks corresponding to three Δ-5 desaturase (D5D) derived fatty acids [20:3n6 (Δ7, 11, 14), 20:4n3 (Δ7, 11, 14, 17) and 22:4n3 (Δ9, 13, 16, 19)] were found in the liver PL fractions of D6KO mice fed LA-rich safflower oil (SF) and LA+ALA-enriched soybean oil (SO) diets (Figure 2; Figure 3). These three fatty acids were absent from all three experimental diets, and were also absent in the chromatographs of WT SF and SO fed mice. All three novel fatty acids were found to be differentially distributed among the six PL fractions (PC, phosphatidylcholine; PE, phosphatidylethanolamine; PS, phosphatidylserine; PI, phosphatidylinositol; SM, sphingomyelin; Lyso-PC, lyso-phosphatidylcholine). The PI fraction contained the highest percentage of 20:3n6 (Δ7, 11, 14) (SO =23.5 + 4.4%; SF = 30.6 + 1.6%) followed by the PS (17.7 + 1.6%), PE (11.7 + 1.7%), SM (8.4 + 3.2%), Lyso-PC (4.8 + 1.0%), and PC factions (4.1 + 0.6%) in SO-fed D6KO mice; and followed by PS (20.9 + 2.2%), PE (13.4 + 0.7%), PC (5.4 + 0.4%), SM (5.0 + 3.7%), and Lyso-PC fractions (4.0 + 1.7%) in SF-fed D6KO mice (Figure 3A). In the PI fraction of D6KO mice fed SF, 20:3n6 (Δ7, 11, 14) comprised almost a third of all fatty acids, and in the PI fraction of SO-fed D6KO mice, this novel fatty acid accounted for almost a fourth of total fatty acids. 20:4n3 (Δ7, 11, 14, 17) and 22:4n3 (Δ9, 13, 16, 19) were also selectively distributed across PL fractions, with the highest levels (relative to total fatty acids) found in PE, PI and PS fractions (Figure 3B,C).

Trace amounts of all three novel fatty acids were found in several PL fractions of menhaden (MD)-fed D6KO mice, but in much lower levels in comparison to D6KO mice fed SO and SF (Figure 3). In the PC fraction of MD-fed mice total D5D fatty acids composed 0.23 + 0.03% of total fatty acids; in comparison, the PC fraction of SF and SO-fed mice contained 5.40 + 0.35% and 4.77 + 0.69%, respectively. Similar trends were observed in other PL fractions (Figure 3).

**Figure 4:**
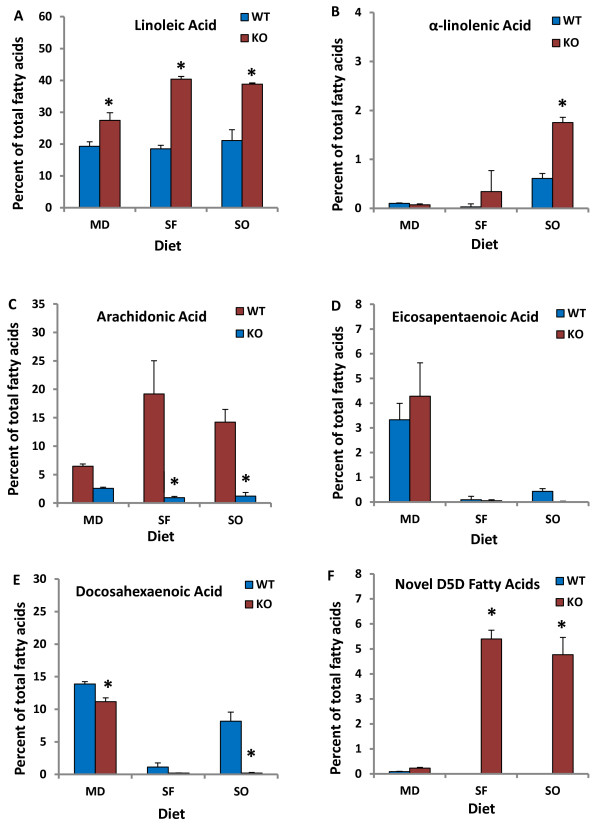
**Comparison of select fatty acid values found in the liver PC fraction (shown as percent of total PC fatty acids) between genotypes and across dietary treatment groups: A) linoleic acid; B) α-linolenic acid; C) arachidonic acid; D) eicosapentaenoic acid; E) docosahexaenoic acid; F) total novel Δ-5 fatty acids.** *indicates significant difference (p < 0.05) between D6KO and WT pairs; differences between means determined by 2-way ANOVA. MD, menhaden oil, SF, safflower; SO, soy.

D5D fatty acids were not detected in WT mice fed SF and SO diets, while PL fractions of WT mice fed MD were all found to contain less than 1% D5D fatty acids. Novel D5D fatty acid content of all PL fractions was significantly different (p < 0.001) between WT and D6KO mice fed either SO and SF, but% D5D fatty acids were similar between WT and D6KO mice fed MD (Figure 4F).

### Comparison of liver LA and ALA levels across treatment groups

LA content increased in most PL fractions of the three D6KO treatment groups. In all PL fractions of SO-fed D6KO mice LA content increased, and this increase was significant in most fractions. In particular, in the PC fraction of SO-mice LA content almost doubled from a mean level of 21.12 + 3.38% in WT mice to 38.83 + 0.34% in D6KO mice (p < 0.0001) (Figure 4A); while in the PE fraction LA content showed a 3-fold increase. A similar effect was seen between D6KO and WT mice fed SF diet (Figure 4A). In contrast, in MD-fed mice, LA content also increased in 5 of the 6 PL fractions, but this increase was less than 2-fold in any fraction.

In all PL fractions of SF-fed and SO-fed mice ALA content remained unchanged or increased. The increase in ALA content was especially evident in SO-fed mice, as the SO diet contains greater levels of ALA than the SF diet. Across all PL fractions of D6KO mice on a SO diet there was an approximately 3-fold or greater increase in ALA content when compared with WT mice fed SO-diets. In particular, the PC fraction of SO-fed D6KO mice contained 1.75 +0.11% ALA compared with only 0.61+ 0.10% in WT mice (p < 0.0001) (Figure 4B). Unlike the results seen in SF and SO fed mice, ALA levels remained constant or showed non-significant decreases in MD-fed D6KO in comparison to their WT counterparts.

### Highly unsaturated fatty acids

Levels of AA were significantly lower in most PL fractions of SF and SO-fed D6KO mice compared with their WT counterparts (Figure 4C). There was also a small decrease in AA content in D6KO mice fed MD compared with WT mice which did not reach significance in most PL fractions.

EPA content remained similar or decreased in D6KO mice fed SO or SF diets, while EPA levels actually increased in MD-fed D6KO mice. After 4 weeks of feeding D6KO mice a soy-based diet, EPA content decreased non-significantly in the PC (Figure 4D) and PE fractions. In the PE fraction of MD-fed D6KO mice, EPA levels increased from 4.76 + 0.23% to 8.89 + 1.07% (p < 0.05), and a similar increase was seen in most other fractions.

There was a decrease in DHA across all PL fractions in both SF-fed and SO-fed D6KO mice. In the PL fractions of MD-fed D6KO mice, DHA content remained equivalent to WT mice, or declined (Figure 4E), but to a lesser extent than was seen between D6KO and WT SF or SO treatment groups.

### N6/N3 ratio

The n6/n3 fatty acid ratio significantly increased 2–6 fold in the PC and PE fractions of SO and SF-fed D6KO mice (SO WT ratio = 4.04 ± 0.74 vs. SO D6KO ratio =15.44 ± 0.56; SF WT ratio = 25.29 ± 11.32 vs. SF D6KO ratio = 69.66 ± 23.71; p = 0.05), while there was only a slight non-significant increase in this ratio in MD-fed mice (MD WT ratio = 1.59 ± 0.18 vs. MD D6KO ratio = 1.89 ± 0.33).

### Novel fatty acid distribution in serum lipid fractions

Serum phospholipid fractions were analyzed and it was determined that all three novel D5D products were present in the PL factions of D6KO mice. The distribution of serum phospholipid D5D fatty acids was similar to the distribution found in liver PL fractions (Table 2).

**Table 2:** Novel D5D product composition in the serum phosphatidylcholine fraction

**Fatty Acid**	**Menhaden**	**Safflower**	**Soy**	**Menhaden**	**Safflower**	**Soy**	**Interaction p- value**
	**n = 4**	**n = 7**	**n = 5**	**n = 4**	**n = 7**	**n = 3**	
	**WT**			**KO**			
**20:3(Δ7,11,14)**	0.1 ± 0.09	0	0	0.1 ± 0.04	4.0 ± 1.74 ^a^	2.6 ± 0.69 ^a^	<0.01
**20:4(Δ7,11,14,17)**	0.1 ± 0.07	0.1 ± 0.08	0.2 ± 0.05	0.1 ± 0.03	0.2 ± 0.06	0.1 ± 0.02	0.23
**22:4(Δ9,13,16,19)**	0	0	0	0	0	0.2 ± 0.05 ^a^	<0.01
**Total D5D Products**	**0.2 ± 0.20**	**0.1 ± 0.08**	**0.2 ± 0.05**	**0.2 ± 0.04**	**4.2 ± 1.80**^ a^	**2.9 ± 0.70**^ a^	<0.01

Note: Data were analyzed by 2-way ANOVA and are expressed as means +/− SD. Means with letters represent a significant difference (p < 0.05) from wild-type values within a row.

## Discussion

### Change in liver lipid composition of D6KO mice fed HUFA-deficient diets

The novel D6KO model has the potential to enhance our fundamental understanding of the role of the essential fatty acids ALA and LA in health and disease. However, careful examination of the phenotype under different experimental conditions is needed in order to ascertain its potential utility and limitations. Previous research has used diets containing n-6 and n-3 fatty acids from soy [17], however, the effect of feeding only an LA-enriched diet on the fatty acid composition of liver phospholipid fractions in D6KO mice is currently unknown. Given that n-6 and n-3 fatty acids share this same pathway, it is important to determine if feeding D6KO mice a diet enriched in LA will result in a different hepatic fatty acid profile than mice fed a diet containing both LA and ALA [17]. Therefore, the first objective of the current study was to characterize the liver phospholipid composition of D6KO mice fed a diet rich in LA but void of ALA and HUFAs.

In agreement with previous studies, three novel fatty acid peaks were present in the phospholipid fractions of D6KO mice. The first two fatty acids were identified as 20:3n6(Δ7, 11, 14) and 20:4n3(Δ7, 11, 14, 17) and are the products of desaturation by D5D [17]. Additionally, a third novel fatty acid, 22:4n3(Δ9, 13, 16, 19) was identified as a product of an elongase acting on 20:4n3(Δ7, 11, 14, 17). A diet rich in LA, such as a diet containing safflower oil, results in the production of 20:3n6(Δ7, 11, 14), with trace quantities of the other two D5D fatty acids in the tissues of D6KO mice. Feeding D6KO mice a diet containing a source of ALA, such as soybean oil, results in the production of all three novel D5D fatty acids, with increased production of the two novel n-3 D5D products compared to a SF diet.

Mean weight gain and food intake was not significantly different among treatment groups, which indicates that mice had roughly equivalent caloric intake. The absence of any gross changes in the health status of D6KO mice may also suggest that, over a short duration, the provision of the parent PUFA LA and ALA may be sufficient to maintain regular biological functioning, even as HUFA stores are depleted. Additionally, the similar fatty acid profile of heterozygous D6KO mice and WT mice suggests that even one functional copy of the FADS2 gene is sufficient to maintain normal fatty acid levels. However, treatment duration was relatively short, and future research is required to determine if D6KO mice will differ from WT mice in response to diets of varied fatty acid composition over a longer time frame.

We confirmed that D6KO mice fed SF and SO diets had considerably less EPA, DHA, and AA in all liver PL fractions, as compared to WT mice. The n-6/n-3 fatty acid ratio was also greatly increased in the liver tissues of D6KO mice on SF and SO diets. The n-6/n-3 ratio is one method of assessing tissue n-3 content relative to n-6 fatty acid content [19]. A high n6/n3 ratio, such as the ~15:1 ratio of the average American diet, has been associated with increased disease risk [20], while decreasing this ratio to the recommended ~4:1 has been shown to protect against disease [21,22]. While FADS2 loss of function is rare in humans, several SNPs in the FADS2 gene have been discovered [23,24].Through the modification of desaturase activity, certain FADS2 SNPs have also been associated with disease risk [25,26]. The results of this study then suggest that a decreased ability to process ALA or LA via FADS2 polymorphisms [27] or loss of function can lead to a higher PUFA ratio, which may increase disease susceptibility. Additionally, future studies can use the D6KO mouse as an suitable model to study FADS2 SNPs which lead to decreased D6D activity.

### The selective distribution of novel fatty acids among phospholipid fractions

The distribution of AA across phospholipid fractions is tightly regulated, with each PL fraction containing varied amounts of this n-6 fatty acid. In agreement with other studies [9,28], we found that PI and PE fractions were enriched in AA (data not shown). 20:3n6(Δ7, 11, 14), the major D5D fatty acid, was shown to be selectively distributed among the PL fractions, composing almost a third of PI fatty acids in SF-fed D6KO mice, and also contributing largely to the PS and PE fatty acid pool. Thus, the distribution of this fatty acid seems to follow a similar distribution to AA. This could be associated with selective PL remodeling seen with AA. For example, in a previous study it was determined that while the initial incorporation of AA into PC was high, over time there was a greater incorporation of AA into PE and PI [9]. This finding, and others [29,30] suggest that some enzymes associated with PL remodeling show specificity for AA. It is plausible that these same enzymes could also have a similar affinity for 20:3n6(Δ7, 11, 14), causing the distribution of this novel n-6 fatty acid to mimic the selective distribution of AA across PL fractions.

Novel D5D fatty acids were also discovered in serum phospholipids of D6KO mice, suggesting that once formed, D5D fatty acids are not retained by the liver, but make their way into the circulation. The effect of these D5D fatty acids on extra-hepatic tissues is currently unknown, but certainly could be a potential confounder.

### A diet supplemented in preformed HUFA partially rescues D6KO phenotype

The MD diet used in this study contained 3% (w/w) menhaden oil and 7% safflower oil, amounting to approximately 0.4% (w/w) DHA, 0.04% AA, and 0.39% EPA (based on% composition values in Table 3). Previous studies have shown that 0.2% w/w DHA (DHASCO) supplementation is sufficient to restore reproductive ability in D6KO mice [15], while 0.4% w/w AA supplementation could prevent the dermatitis and intestinal ulcers [17] otherwise seen in D6KO mice fed HUFA deficient diets. Whether the provision of only AA, EPA/DHA, or both n-6 and n-3 HUFA is sufficient for optimal rescue in these disease models requires further study. Towards understanding this important question, this study demonstrates the efficacy of menhaden oil, a natural oil containing a mixture of n-6 and n-3 HUFA, in rescuing the D6KO phenotype. This finding is highly relevant and demonstrates the utility of menhaden oil as an important control group in future investigations using this model.

**Table 3:** Diet fatty acid composition

**Fatty Acid**	**10% Safflower**	**10% Soy**	**3% fish + 7% safflower**
	**n-6 (LA)**	**n-3 (ALA)**	**n-3 (HUFA)**
**14:0**	0.2	0.2	2.6
**16:0**	6.3	10.8	10.4
**16:1**	0.1	0.2	3.2
**18:0**	2.6	4.3	3.0
**18:1c9**	14.6	21.1	12.2
**18:1c11**	0.7	1.5	1.4
**18:2n6**	73.6	52.8	53.7
**18:3n6**	0.0	0.0	0.1
**18:3n3**	0.2	6.9	0.6
**18:4n3**	0.0	0.0	1.1
**20:0**	0.4	0.4	0.4
**20:1**	0.1	0.3	0.2
**20:2n6**	0.0	0.0	0.2
**20:3n6**	0.1	0.0	0.1
**20:4n6**	0.0	0.0	0.4
**20:3n3**	0.0	0.0	0.1
**20:5n3**	0.0	0.0	3.9
**22:0**	0.3	0.6	0.4
**22:1**	0.1	0.2	0.1
**22:4n6**	0.0	0.0	0.1
**22:5n6**	0.0	0.0	0.2
**22:5n3**	0.0	0.1	0.8
**24:0**	0.2	0.2	0.1
**22:6n3**	0.0	0.0	4.0
**24:1**	0.0	0.0	0.3
**sum**	100%	100%	100%

Data are the means of analysis in duplicate and represent percentage of total fatty acids in each diet.

The addition of menhaden oil to the diet of D6KO mice had a profound effect on PUFA levels in the PL fractions of the liver. In contrast with D6KO mice on SF or SO diets, D6KO mice fed MD diets had liver lipid profiles that resembled their WT counterparts. While the PC fraction of MD-fed D6KO mice did contain significantly more LA compared with their WT counterparts, the difference in LA content was much greater between WT and D6KO mice in the SO and SF treatment groups.D6KO mice fed MD had EPA levels which exceeded levels found in WT mice, while DHA levels approached WT levels. ALA levels in MD-fed D6KO and WT mice were nearly equivalent.

MD-fed D6KO mice also showed similar novel D5D fatty acid profiles to the MD WT treatment group, possibly through the action of HUFA to stimulate and inhibit certain transcription factors. HUFA are well-known inhibitors of SREBP-1c (sterol regulatory element binding protein-1c) which increase the transcription of D6D [1,31,32] and D5D [33]. Studies have also concluded that during EFA deficiency or starvation, PPARα (peroxisome proliferator-activated receptor alpha) is activated through the endogenous release of PUFA and up-regulates the transcription of D6D and D5D [33,34]. Thus, in conditions where HUFA are restricted, SREBP-1c and PPARα,-two otherwise opposing enzymes- may act to increase the transcription of D5D, resulting in increased production of D5D fatty acid products. While gene expression was not specifically evaluated in the current study, the study results support this hypothesis of D6D and D5D regulation by PPARα and SREBP-1c, as the hepatic PC fraction of MD-fed D6KO mice contained less than 0.25% novel D5D fatty acids (of total fatty acids), while SO and SF-fed D6KO mice PC fractions contained between 4-6% novel fatty acids.

## Conclusion and future directions

The present study demonstrates that the conversion of LA and ALA toAA, EPA, and DHA is abolished in D6KO mice. This is readily observed by the presence of three novel D5D fatty acids in both liver and serum phospholipid fractions. However, since the function of these D5D fatty acids is unknown, their unwanted production in D6KO mice represents a limitation of this mouse model. The implications of this study demonstrate that this limitation can be overcome through the provision of dietary HUFAs, which inhibit the D5D metabolism of ALA and LA. The major dietary n-3 s and n-6 s consumed in a Western diet are ALA and LA, yet minimal conversion of these fatty acids into their long chain metabolites occurs in humans. Little is known about the independent biological actions of LA and ALA, thus with the aid of a fully characterized D6KO model, future dietary studies can provide better insight into the independent biological roles of LA and ALA in health and disease.

## Methods

### Animals and diet

Heterozygous D6KO mice backcrossed to a C57BL/6 background [35], were transferred from University of Illinois at Urbana-Champaign to the University of Guelph where a colony of heterozygous mice was established. Experimental mice consisting of D6KO and WT mice were obtained from the breeding of harems consisting of one heterozygous male and 2–3 females. Breeders were fed standard laboratory mouse chow (Harlan Tekad #9004), and pups were continued on this diet until 28 days old. Mice were housed within ventilated cages at 22°C in humidity-controlled environment on a 12 hour light: 12 hour dark cycle for the study duration.

At 21 days mice were weaned, and tail snips were obtained for DNA extraction and PCR analysis to determine genotype [17]. PCR primers D6D WT forward (CGGTGGGAGGAGGAGTAGAAGAC); D6D WT reverse (CCTCTCCCTGGTTACCTCCCTTC); D6D KO forward (GCTATGACTGGGCACAACAG); and D6D KO reverse (TTCGTCCAGATCATCCTGATC) were used.

Twenty-eight-day old homozygous D6KO and WT mice were placed on one of three experimental diets (Table 3; modified AIN93G diet; Research Diets): 10% Soy diet (SO; cat# D09072305) (n = 5 WT and 3 KO), 10% safflower diet (SF; cat# D04092701) (n = 7 WT and 7 KO), or 3% menhaden + 7% safflower diet (MD; cat# D04092703) (n = 4 WT and 4 KO). Mice were fed experimental diets for 28 days. At 56 days of age, the experimental mice were euthanized using CO_2_, and final body weights recorded. This investigation was approved by the University of Guelph Animal Care Committee in accordance with the requirements of the Canadian Council on Animal Care.

### Fatty acid analysis

Upon termination, liver samples were collected, flash frozen in liquid nitrogen, and stored at −80°C. For analysis, 0.1 g of liver tissue was weighed and homogenized in 2.5 ml of 0.1 M potassium chloride. 10 ml chloroform (Fisher, Cat #C298-4): methanol (Fisher, Cat #A452-4) solution (2:1 v/v) was then added to the homogenized liver samples, according to Folch *et al*[36]. Samples were vortexed, flushed with nitrogen to prevent oxidation, and stored at 4°C overnight [37]. Samples were then centrifuged at 577xg for 10 minutes to separate phases. The lipid-containing chloroform layer was extracted into a pre-weighted acid-washed screw cap test tube with Teflon lined cap, dried down under nitrogen gas, and weighed to determine the total mass of lipids. 6 ml chloroform was added back into homogenized liver sample, and the extraction process was repeated. Dried lipid samples were reconstituted at 10 mg/ml chloroform for thin layer chromatography (TLC).

Phospholipid fractions were then separated using TLC. Briefly, silica H plates (VWR 5721–7) were activated by heating at 100°C for 1 hour. 100μl of each liver lipid sample were spotted along 2 cm lanes scored onto the activated plates. Samples were run alongside known phospholipid standards for 2.5 hours in chloroform/ methanol/ 2-propanol (Fisher A416-1) /KCl (0.25%w/v)/triethylamine (Sigma Cat# CT0886) (30:9:25:6:18). Fractions were visualized using 0.1% 8-anilino-1-naphthalene sulfonic acid (w/v) (Fluka # 10417) under UV light. Bands corresponding to PC, PE, PS, PI, SM and lyso-PC were identified and individually scraped into acid washed test tubes already containing 1.0μl (PC) or 0.1μl (PE, PS, PI, SM, Lyso PC) 17:0 FFA as an internal standard and 2 ml hexane. 2 ml 14% boron triflouride-methanol (Sigma, cat #B1252) was next added, and samples were methylated for 1.5 hours at 100C. Samples were allowed to cool (RT), then 2 ml distilled water was added to each sample to halt methylation, and samples were centrifuged at 577 × g for 10 minutes. The hexane layer containing methylated fatty acids was extracted into a gas chromatography vials. Samples were then dried down under a gentle stream of nitrogen gas and reconstituted in in 30μl (100μl for PC) hexane in glass gas chromatography inserts [37].

PL fatty acid methyl esters were quantified using an Agilent 6890 N gas chromatograph equipped with a flame ionized detector and separated on a Supelco SP-2560 fused silica capillary column (100 m, 0.2 μm film thickness, 0.25 mm i.d.; Sigma cat #24056 ) [37]. Hydrogen was used as the carrier gas and set at a constant flow rate of 30 mL/min. Samples were injected in splitless mode. The injector and detector ports were set at 250°C. Fatty acid methyl esters were eluted through the column using temperature program of: 0.2 min at 60°C, then increasing 13°C/min until a temperature of 170°C was reached. 170°C was held for 4 min, then increased 6.5°C/min to 175°C, increased 2.6°C/min to 185°C, increased 1.3°C/min to 190°C, and finally increased 13°C/min to 240°C and held at this temperature for 13 min. The run time per sample was 37.77 min. Fatty acids were identified by comparing peak retention times with those of a known standard (Nu-Chek-Prep, Elysian, MN). Fatty acid peak areas were determined using EZChrom Elite software (Version 3.3.2). Fatty acid values were expressed as percent area in each PL fraction.

Serum lipid fraction analysis was carried out under the same conditions as liver tissue samples above using 50 μl serum. Briefly, serum lipids were double extracted, dried down and reconstituted in 150 μl chloroform. 100 μl of reconstituted lipid sample was spotted onto an activated TLC H-plate, and phospholipid bands were scraped, methylated for an hour, and analyzed using Agilent 7890 gas chromatograph under the same temperature program as liver samples.

### Statistical analysis

Data were analyzed using SAS (v. 9.1) statistical software. 2-way analysis of variance test was performed on data, followed by Tukey post-hoc tests on main effects when the interaction term was not significant; Least Squares Means was used when interaction values were significant. Statistical significance was set at p < 0.05.

## Abbreviations

D6D, Delta-6 desaturase; D6KO, Delta-6-desaturase knock out; WT, Wild type; HUFA, Highly unsaturated fatty acids; PUFA, Polyunsaturated fatty acid; D5D, Delta-5 desaturase; LA, Linoleic acid; ALA, Alpha-linolenic acid; SF, Safflower oil; SO, Soybean oil; MD, Menhaden oil; AA, Arachidonic acid; EPA, Eicosapentaenoic acid; DHA, Docosahexaenoic acid; PL, Phospholipid; PC, Phosphatidylcholine; PE, Phosphatidylethanolamine; PS, Phosphatidylserine; PI, Phosphatidylinositol; SM, Sphingomyelin; Lyso-PC, Lyso-phosphatidylcholine; TLC, Thin layer chromatography.

## Competing interests

DM has received funding from the Canola Council of Canada. All other authors declare that they have no competing interests.

## Authors’ contributions

(All authors read and approved the final manuscript). JM performed liver lipid analysis, analyzed data, and drafted the manuscript. FL performed liver lipid analysis and analyzed data. MM contributed to the design of the study. AR performed serum lipid analysis and analyzed data. MHM contributed to the design of the study. MTN generated the mouse model used in the study. DWLM contributed to the conception and design of the study, supervised the project and edited the manuscript.
